# Perception of social inequities in the access to the kidney transplant waiting list by nephrology trainees: a national survey

**DOI:** 10.1186/s12882-022-03017-w

**Published:** 2022-12-08

**Authors:** Lucile Paris, Eve Calvar, Rémy Morello, Angélique Lecouf, Mathilde Beaumier, Thierry Lobbedez, Valérie Châtelet

**Affiliations:** 1 Centre Universitaire des Maladies Rénales, CHU de Caen, Avenue de la Côte de Nacre, 14 033 Caen Cedex 9, France; 2 Normandie Université, Unicaen, UFR de Médecine, 2 rue des Rochambelles, 14032 Caen Cedex, France; 3 U1086 INSERM – ANTICIPE – Centre Régional de Lutte Contre le Cancer, François Baclesse, Caen, France; 4 Plateforme de Méthodologie, CHU de Caen, Avenue de la Côte de Nacre, Niveau 3, CS 30001, 14033 Caen Cedex 9, France

**Keywords:** Access to kidney transplant waitlisting, Nephrology trainees, Perception, Social inequalities in health

## Abstract

**Background:**

Social inequalities in health are responsible for disparities in access to the kidney transplant waiting list (KTWL). The perception of disparities by nephrologists has consequences for the registration on the KTWL. The purposes of our study were to assess the perception of the factors implicated in the disparities in access to the KTWL by nephrology trainees and to assess the quality of the questionnaire.

**Methods:**

A questionnaire was developed to assess the perception of the determinants of the inequities in access to waitlisting. Continuous variables were described by median, 1st and 3rd quartiles. Categorical variables were described by frequencies and percentages. A principal component analysis and a hierarchical cluster analysis were performed to approach the correlation between the variables. A scree plot and a factor analysis were performed to determine the dimensions of the questionnaire. The internal consistency was estimated by Cronbach’s coefficient.

**Results:**

The response rate was 98/110 (89%). The determinants of inequities in the access to KTWL not perceived by the nephrology trainees were “female sex”, “income level” and “the centre provision to adapt the information to all of the patients” (18,3%, 36,7, 47% respectively). “Age”, “being born abroad”, “place of living”, “education level”, “transplant centre”, “the health care provider” were determinants of disparities perceived by most of the trainees (85,7%, 75,5%, 82,6%, 78,6%, 73,5% et 78,5% respectively). Items related to the transplant centre were positively correlated, as well as “being born abroad”, “education level” and “income level”. The Cronbach’s coefficient was 0,60.

**Conclusion:**

Social inequalities in health are partially perceived by nephrology trainees. A teaching session could raise nephrologists’ awareness of this issue and could help reduce the impact of these disparities on the course of ESKD (end-stage kidney disease) patients.

**Supplementary Information:**

The online version contains supplementary material available at 10.1186/s12882-022-03017-w.

## Introduction

It is widely accepted that compared to dialysis, kidney transplantation is associated with a lower risk of mortality, a greater quality of life, and lower health care costs [1, 2]. Access to kidney transplant waiting list must be promoted among end-stage kidney disease (ESKD) patients without any medical contraindication who have the willingness to be transplanted. It is recognized that efforts must be made to act against inequalities in health that are socially determined, depend on individual, environmental and societal conditions and affect access to kidney transplantation [3].

In high-income countries, there are disparities in access to the KTWL due to geographic, demographic and socioeconomic factors [3, 4]. It is also well known that there are important disparities between countries, as in many low-income countries there is no or very difficult access to kidney transplantation [5]. Health care professionals (HCPs) play a key role in providing patients information about kidney transplantation for shared decision-making, guidance along the transplantation course and support for registration on the KTWL.

Acting against social inequities implies that HCPs have the perception that social disparities affect access to the KTWL. To the best of our knowledge, only a few studies conducted in the United States have focused on this topic [6–9]. No study has investigated the perception of nephrology trainees of social inequities in waitlisting, which is of importance, as this topic could be implemented in a medical education program.

The purpose of our study was to determine if nephrology trainees perceive that social disparities could influence access to the KTWL. The other objective of our study was to assess the quality of the questionnaire that was designed to address this question.

## Materials and methods

### Study population

In France, the nephrology curriculum is divided into different parts. During the first year, trainees learn basic nephrology knowledge. During the following years, advanced insights into nephrology are provided.

The study population was the 110 nephrology trainees in their 3rd or 4th year of internship from 28 teaching hospitals in France (including French overseas departments). Of the 110 questionnaires, 98 questionnaires were completed by the trainees. The questionnaire was distributed and returned during a national teaching session in March 2021.

### Development of the questionnaire

The determinants of the inequities in access to the KTWL were identified from an extensive literature review [10–12]. A self-completed questionnaire (see Supplementary Fig. 1, Additional file 1) was developed to assess the perception of the respondent regarding patients’ and centre’s determinants of inequities in waitlisting. In addition, 5 questions were added to anonymously collect information about the respondents.

There were 12 items to explore the perception of 15 factors of social inequities in access to waitlistings related to patients’ socioprofessional characteristics and the organization of medical centres.

A Likert scale with 6 response modalities was used to measure the perception of 11 out of 12 questions. For each item, the questionnaire included quantitative answers from 0 to 5: do not know (0), strongly disagree (1), disagree (2), undecided (3), agree (4), and strongly agree (5). The questionnaire allowed the trainees to write a comment regarding each question. The questionnaire also included a multiple-choice question to assess the perception of the nephrology trainees regarding the role of specific categories of persons in gaining access to the KTWL.

The questionnaire was previously tested in 2021 in a pilot study (not published) conducted on 112 HCPs from 8 dialysis centres in our area. This pilot study was properly understood by the HCP, and there was no difficulty answering that led to major modification of the items.

### Studied variables

The characteristics of the respondents collected were gender, age, semester of internship, university hospital centre and the principal field of interest among nephrology, dialysis and transplantation.

Determinants of inequity in access to the KTWL studied were gender, age, being born abroad, living place, education level, and income level. Perceptions about the effect of the centre’s characteristics were also collected: centre provision to adapt the information, role of the transplant centre and of the HCP in access to the KTWL. The question about the effects of specific categories of persons included 6 modalities: unemployment, physical disability, mental disability, addiction, “stay-at-home” parent and pensioner.

### Statistical analyses

#### Results of the survey

Continuous variables were described by median, 1st and 3rd quartiles. Categorical variables were described by frequencies and percentages.

#### Questionnaire assessment

The quality of each item was assessed with a graphical representation (barplot) to detect a floor or a ceiling effect of the answer. In addition, to approach the correlation between the items of the questionnaire with a graphical representation, a principal component analysis (PCA) was carried out. Hierarchical clustering was also conducted to explore the relationship between the items.

To evaluate the number of dimensions, a questionnaire analysis was performed with a scree plot and a factor analysis [13]. A scree plot with 20 simulations was used to evaluate the number of dimensions of the questionnaire items. A factor analysis was carried out to determine which items belonged to which questionnaire’s dimension. The results were expressed with coefficients with a value from − 1 to 1.

Cronbach’s α coefficient was estimated to explore the internal consistency of the questionnaire.

Statistical analysis was performed with R 4.2.0 (R Foundation for Statistical Computing) using psych packages.

### Missing data

Among the 98 questionnaires, there were 14 questionnaires with missing data. Most of the missing data concerned respondent variables (8 questionnaires). There was less than 10% missing data; consequently, a complete case analysis was performed.

## Results

### Results of the survey

#### Characteristics of the trainees

Ninety-eight nephrology trainees were included in this study. The sex ratio was (M/F) 55/43, and the median age was 27 years (median = 27, 1st quartile = 27, 3rd quartile = 28).

Most of the nephrology trainees were in their 6th or 8th semester of internship 83/96 (84.7%).

Of the 92 that responded to this item, 28 (28.6%) answered that transplantation was their main field of interest, while 64/92 (65.3%) answered that they were interested in nephrology and/or dialysis.

#### Perception of the role of the determinants in the inequity in access to the kidney transplant waiting list

The respondents did not perceive that sex had an effect on access to waitlisting (male: 64/98 (65.3%), female: 56/98 (57.1%) of disagree or strongly disagree). A large proportion were neutral on the role of sex (male: 21/98 (21.4%), female: 20/98 (20.4%)). Likewise, nephrology trainees did not perceive the income level (40/98 (40.8% disagreed)) or the centre provision to adapt its information to all patients (47/98 (48% agreed)) as barriers to access to waitlisting. The results of the survey are displayed in Table 1.

**Table 1:** Results of the survey

Items Number (%)	0	1	2	3	4	5	Missing data
**Male sex**	5 (5.1)	22 (22.4)	42 (42.9)	21 (21.4)	7 (7.1)	1 (1)	0 (0)
**Female sex**	4 (4.1)	20 (20.4)	36 (36.7)	20 (20.4)	17 (17.3)	1 (1)	0 (0)
**Age**	0 (0)	2 (2)	6 (6.1)	6 (6.1)	56 (57.1)	28 (28.6)	0 (0)
**Born abroad**	2 (2)	5 (5.1)	10 (10.2)	7 (7.1)	53 (54.1)	21 (21.4)	0 (0)
**Living place**	1 (1)	1 (1)	5 (5.1)	9 (9.2)	51 (52)	30 (30.6)	1 (1)
**Education level**	0 (0)	2 (2)	12 (12.2)	7 (7.1)	58 (59.2)	19 (19.4)	0 (0)
**Income level**	6 (6.1)	15 (15.3)	25 (25.5)	16 (16.3)	29 (29.6)	7 (7.1)	0 (0)
**Centre provision to adapt the information**	5 (5.1)	3 (3.1)	23 (23.5)	20 (20.4)	39 (39.8)	8 (8.2)	0 (0)
**Transplant centre**	4 (4.1)	1 (1)	6 (6.1)	12 (12.2)	52 (53.1)	20 (20.4)	3 (3.1)
**Health care professional**	1 (1)	0 (0)	6 (6.1)	11 (11.2)	60 (61.2)	17 (17.3)	3 (3.1)
**Others**	14 (14.3)	0 (0)	1 (1)	27 (27.6)	36 (36.7)	15 (15.3)	5 (5.1)

Ninety-eight questionnaires were completed out of 110 questionnaires distributed

11 items were answered using the Likert scale of 6 points: don’t know (0), strongly disagree (1), disagree (2), undecided (3), agree (4), strongly agree (5)

The respondents identified some of the patient’s characteristics as factors implicated in restricted access to the KTWL: age, being born abroad, living place and education level (84/98 (85.7%), 74/98 (75.5%), 81/98 (82.6%) and 77/98 (78.6%) of the trainees agreed and strongly agreed, respectively) (Table 1). They felt that the transplant centre and the HCP could influence access to the KTWL (72/95 (73.5%) and 77/95 (78.5%), respectively). Regarding the question about other possible factors that could hinder access to waitlisting, 51/93 (52%) agreed, only 1/93 (1%) disagreed.

Concerning the perception of specific categories at risk of restricted access to the KTWL, the trainees did not notice that unemployment, being retired or being a “stay-at-home” parent hindered access to waitlisting (71/98 (72.4%), 93/98 (94.9%), 91/98 (92.9%), respectively). Most of them answered that suffering from mental disability, addiction or physical disability were conditions that limited access to waitlisting (92/98 (93.9%), 93/98 (94.9%) and 58/98 (59.2%)).

### Questionnaire assessment

#### Distribution of the items

The graphical representation of the answers of each item did not show a ceiling effect nor a floor effect (see Supplementary Fig. 2, Additional file 2).

#### Correlation between the items

In the correlation matrix, the pair of variables “male sex” and “female sex” were correlated. The variable “female sex” was used in further analysis to reflect the role of sex in the inequity in the access to waitlisting. The principal component analysis and the cluster analysis were consistent (see Supplementary Fig. 3, Additional file 3, and Fig. 4, Additional file 4) showing that the variables “income level” and “education level” positively correlated. The variable “born abroad” can be aggregated into this cluster. The variables “health care professional” and “transplant centre” were also correlated.

#### Scree plot and factor analysis

The scree plot showed that there were two dimensions (see Supplementary Fig. 5, Additional file 5), in the factor analysis (Table 2), Factor 1 had the strongest association with all of the items concerning the patient factors (age, female sex, born abroad, living place, education level and income level). Three items had large loadings on Factor 1: “born abroad”, “education level” and “income level”. Therefore, factor 1 could be labelled as dimension of the patient’s characteristics. Factor 2 was associated with the transplant centre’s characteristics: “centre provision to adapt the information”, “transplant centre” and “health care professional”.

**Table 2:** Results of the factor analysis

Items	Factor 1	Factor 2
**Female sex**	0,276	−0,010
**Age**	0,329	−0,315
**Born abroad**	0,653	−0,122
**Living place**	0,244	0,111
**Education level**	0,587	0,190
**Income level**	0,657	0,313
**Centre provision to adapt the information**	−0,244	0,128
**Transplant centre**	0,077	0,585
**Health care professional**	0,049	0,481

#### Cronbach’s alpha

The Cronbach coefficient, which estimates the internal consistency of the questionnaire, was 0. 60.

## Discussion

It has been demonstrated that barriers at the patient level (sex, age, socioeconomic status) and related to medical organizations could lead to social inequities in access to the KTWL [10–12]. HCPs in charge of ESKD patients have a critical role during the transplantation care continuum since they must help individuals at the time of registration, performing the required medical and psychosocial workup, assessing the patient’s willingness to be transplanted and ensure that the transplantation process and follow-up are fully understood. HCP perception of the causes of inequities in access to KTWL may help ESKD subjects be registered on the list regardless of their age, sex or socioeconomic status. Education sessions dedicated to HCP could limit their misconceptions, in support of this it has been shown that training programs for HCP improve the perceptions and skills of the HCP in palliative care [14].

Among the factors of limited access to the KTWL, age is the one most well perceived by the nephrology trainees; nevertheless, a significant proportion of trainees did not notice that age was a cause of disparities in the patient’s care. The role of the patients’ age, even adjusted on comorbidities, on access to the KTWL was documented in several studies [11, 15–17]. In one French study, patients older than 70 years old had a lower access to KTWL than their counterparts in the reference group of 18 to 39 years [18]. Interestingly, it has been shown that patients older than 65 years were more likely not to have had discussions with HCPs about a kidney transplant as a treatment option [19].

Our study also underlines the fact that nephrology trainees were unaware of sex disparities in access to the KTWL, although sex inequity was identified in many studies [10, 11, 15, 20–22]. A French study by Couchoud et al. [23] also demonstrated that women had a longer time from dialysis start to registration than men. Segev et al. [24] showed that women had less access to transplantation than men and that there was an interaction between sex and age. Their assumption was that older and sicker women were probably more likely misclassified (or they misclassified themselves) as too frail to undergo a kidney transplant. In contrast, Sheikh et al. [25] demonstrated that women were more compliant and had equal or better outcomes after transplant than men. A study conducted in the US highlighted the fact that women undergoing haemodialysis were less likely than men to have discussions about kidney transplantation as a treatment option [19]. The results of our study are consistent with the results of an American study that showed that the dialysis staff were unaware of sex transplant disparities [9].

Being born abroad was not perceived as a factor of unequal access to kidney transplant by 25% of nephrology trainees. Numerous studies have investigated racial disparities in access to kidney transplantation [10, 17, 26]. Harding et al. [4], Patzer and McClellan [27] and Patzer et al. [28] documented that racial disparities were strongly associated with socioeconomic status and independently associated with each step of the pathway from registration to transplantation. Hamoda et al. [8] suggested that medical mistrust, previously experienced discrimination and perceived racism were associated with a decreased evaluation for transplantation initiation. In France, the median waiting time for patients from Sub-Saharan Africa were more than twice the median waiting time for patients from mainland France [29]. A recent study suggested that removing racial coefficients from formulas to estimate the glomerular filtration rate would decrease racial disparity in access to preemptive kidney transplant [30]. Language barriers may also affect waitlisting registration, as suggested in a recent study from the US [31].

Living place was identified as a barrier to waitlisting by most of the trainees. In France, Pladys et al. [18] showed that access to the KTWL was higher in Ile-de-France than in the other regions. Geographical variation in access to the KTWL was also described in the US with rates ranging from 37% lower to 64% higher than the national average. States with lower waitlisting rates had higher transplant rates [32]. In a study from the US, there was no significant difference between patients living in a distant area from the transplant centre compared to the other patients regarding the waiting time to kidney transplantation [33].

Education level was recognized as a factor of inequity in access to the KTWL by most of the trainees. In the literature, a French study highlighted that the more educated the patients were, the greater they were stakeholders in their medical care in nephrology [34]. A survey conducted by REIN suggested that transplanted patients were more educated than patients not registered on the KTWL [3].

Although well described in the literature [10, 11, 35], the respondents of our study did not perceive income as a cause of disparities in access to the KTWL. Patzer et al. [27] showed that low socioeconomic status contributed to poor health outcomes throughout the patient’s course. Low SES was associated with a reduced access to kidney transplantation. In addition, in the US, patients from disadvantaged neighbourhoods had lower access to kidney transplants [36]. In the French northwest area, preemptive registration was lower in the most deprived population [37]. Interestingly, most of the respondents did not think that specific categories exposed to social deprivation (unemployment, pensioner, “stay-at-home” parent) had limited access to registration, whereas they have been shown to have limited access in the literature [28].

Most of the respondents identified that patients suffering from addiction or mental disability had less access to KTWL. These two categories of patients have been cited as relative contraindications to kidney transplantation [3].

The trainees did not perceive that centre provision to adapt the information to all patients could be a factor in unequal access to waitlisting. Taylor et al. [38] showed that CKD patients often have limited health literacy. It has been suggested that limited health literacy hinders access to kidney transplantation [39]. Morony et al. [40] noticed that written information resources intended for CKD patients must be adapted to their level of health literacy.

Our study shows that trainees were aware of the responsibility of both the HCP and the transplant centre in unequal access to waitlisting. In France, being followed in a centre performing transplantation was associated with a higher rate of registration on the KTWL [41]. Conversely, a study performed in England and Wales did not find a difference in the registration rate between centres with a transplant unit and other centres [12].

A review published by Tong et al. [42] in 2014 suggested that nephrologists’ attitudes towards patients’ access to kidney transplantation, was related to the willingness of maximizing efficiency, justifying gains, and protecting unit outcomes.

A study from the US focused on dialysis staff perceptions of barriers to kidney transplantation [7]. Staff identified the patients’ characteristics that contributed to restricted access to KTWL but not the institutional and individual biases. A majority of HCP perceived financial issues and low social support as causes of the African American and sex disparities. It showed that elderly disparities were related to comorbidities, age and finances. According to another American study, the perception of racial disparities in KTWL was lower in HCPs working in low waitlisting dialysis facilities. The HCPs surveyed in this study were mostly white, male and of higher economic status [6]. A study conducted by Tong showed that nephrologists perceived the sex disparity in access to care and identified some barriers, such as socioeconomic disadvantage, lack of support and stereotyping by clinicians [9].

Training programme should be implemented to reduce trainees preconceived misconceptions. To the best of our knowledge, there is no study describing educational programme about this topic. Interestingly, a study showed that a “novel educational intervention” was effective to initiate trainees to goals of care conversations in palliative care [43]. One may hypothesize that to provide a basic knowledge about social inequities trainees could attend didactic session and receive articles on this topic. Given the effectiveness of simulation learning, particularly in nephrology [44, 45], it would be interesting to develop such a program of teaching session. It has been suggested that an interprofessional education simulation programme that focused on a transgender standardized patient could raise awareness about the health disparities faced by transgender individuals [46]. In order to assess the improvement of the perception of the trainees about social inequalities in the access to the KTWL, the survey should be repeated after the implementation of the education programme.

The questionnaire assessment suggested that the questionnaire had 2 dimensions, one related to the patient’s characteristics, while the second was related to the centre’s characteristics. It would be interesting to use this questionnaire to assess not only trainees but also HCP perception of the inequality in registration. One of the limitations of our study is the small size of the population which was not extended to all nephrology trainees in France. However, we believe that trainees in their last years of internship were more able to complete the questionnaire in a relevant way. The lack of internal consistency of the questionnaire was probably partly due to its two-dimensionality, whereas it is admitted that the heterogeneity in item content may challenge the random nature of content sampling [47]. Another limitation of this study is declaration bias, which is inherent to the study design. Another approach to answering the question of the interns’ perception of health inequities would have been to perform a qualitative survey. A strength of this study is the high response rate due to the survey conditions.

## Conclusion

Our study suggests that the perception of inequities in access to the KTWL is partial among nephrology trainees in France. This finding is a matter of concern if one wants to increase access to kidney transplantation.

These findings suggested that teaching sessions should be implemented in the nephrology curriculum to improve nephrology trainees’ knowledge of this topic. We also believe that the survey should be performed on a wider population of HCPs in charge of ESKD patients and after a training program to assess the evolution of their perception after teaching session.

## Supplementary Information

**Additional file 1.** Unvalidated English version of the french questionnaire.

**Additional file 2.** Graphical representation of the item responses.

**Additional file 3.** Principal component analysis.

**Additional file 4.** Hierarchical clustering.

**Additional file 5.** Scree plots.

## Data Availability

The data that support the findings of this study are not publicly available, but they are available from the corresponding author Valérie Châtelet.

## Abbreviations

CKD: Chronic kidney disease
ESKD: End stage kidney disease
HCPs: Health care professionals
KTWL: Kidney transplant waiting list
PCA: Principal component analysis
REIN: Réseau Épidémiologique et Information en Néphrologie
US: United States
