# Supplementary material for: Perception of social inequities in the access to the kidney transplant waiting list by nephrology trainees: a national survey
Source: BMC Nephrol. 2022 Dec 8;23:394. doi: 10.1186/s12882-022-03017-w (PMC9733200; doi:10.1186/s12882-022-03017-w)
Supplement: Supplementary file 1 — Additional file 1. Unvalidated English version of the french questionnaire. [file 12882_2022_3017_MOESM1_ESM.pdf]

Additional file 1. Unvalidated English version of the french questionnaire

- Sex:
- Age:
- Semester of internship:
- University hospital centre:
- Your main area of interest in nephrology:

☐ Nephrology ☐ Dialysis ☐ Transplantation

QUESTIONNAIRE

1/In the French health system, according to you, is male gender of the recipient a factor of inequality in the access to the kidney transplant waiting list?

☐ 1 = Strongly disagree
☐ 2 = Disagree
☐ 3 = Undecided
☐ 4 = Agree
☐ 5 = Strongly agree
☐ 0 = Don't know

2/In the French health system, according to you, is female gender of the recipient a factor of inequality in the access to the kidney transplant waiting list?

☐ 1 = Strongly disagree
☐ 2 = Disagree
☐ 3 = Undecided
☐ 4 = Agree
☐ 5 = Strongly agree
☐ 0 = Don't know

If you want to add more details to your answer, please mention them:
………………………………………………………………………………………………
…………………………………………

3/According to you, is age a factor in inequality in the access to the kidney transplant waiting list?

☐ 1 = Strongly disagree
☐ 2 = Disagree
☐ 3 = Undecided

- ☐ 4 = Agree
- ☐ 5 = Strongly agree
- ☐ 0 = Don't know

If you want to add more details to your answer, please mention them:
………………………………………………………………………………………………
……………………………………………

4/Can being born abroad be taken into account in the inequalities of registration on the kidney transplant waiting list in France?

- ☐ 1 = Strongly disagree
- ☐ 2 = Disagree
- ☐ 3 = Undecided
- ☐ 4 = Agree
- ☐ 5 = Strongly agree
- ☐ 0 = Don't know

If you want to add more details to your answer, please mention them:
………………………………………………………………………………………………
……………………………………………

5/Do you think that the place of residence of the patient is a factor of inequality in the access to the kidney transplant waiting list?

- ☐ 1 = Strongly disagree
- ☐ 2 = Disagree
- ☐ 3 = Undecided
- ☐ 4 = Agree
- ☐ 5 = Strongly agree
- ☐ 0 = Don't know

If you want to add more details to your answer, please mention them:
………………………………………………………………………………………………
……………………………………………

6/In your opinion, can the education level be a factor of unequal access to kidney transplant?

- ☐ 1 = Strongly disagree
- ☐ 2 = Disagree
- ☐ 3 = Undecided
- ☐ 4 = Agree
- ☐ 5 = Strongly agree
- ☐ 0 = Don't know

If you want to add more details to your answer, please mention them:
………………………………………………………………………………………………
……………………………………………

7/In your experience, can the following categories of people have restricted access to the kidney transplant waiting list? Check one or more answers.

- ☐ Unemployment
- ☐ Person with physical disability
- ☐ Person with mental disability
- ☐ Persons suffering from addiction
- ☐ Stay-at-home patient
- ☐ Pensioner
- ☐ Other:
- ☐ 0 = Don't know

If you want to add more details to your answer, please mention them:
………………………………………………………………………………………………
…………………………………………

8/Do you think that the patient's income level can influence access to registration on the kidney transplant waiting list?

- ☐ 1 = Strongly disagree
- ☐ 2 = Disagree
- ☐ 3 = Undecided
- ☐ 4 = Agree
- ☐ 5 = Strongly agree
- ☐ 0 = Don't know

If you want to add more details to your answer, please mention them:
………………………………………………………………………………………………
…………………………………………

9/In your centre, do you think you have the means to adapt your information to all patients concerning registration on the kidney transplant waiting list?

- ☐ 1 = Strongly disagree
- ☐ 2 = Disagree
- ☐ 3 = Undecided
- ☐ 4 = Agree
- ☐ 5 = Strongly agree
- ☐ 0 = Don't know

If you want to add more details to your answer, please mention them:
………………………………………………………………………………………………
…………………………………………

10/In your opinion, is the transplant centre caring for the patient a factor of unequal access to registration on the kidney transplant waiting list?

- ☐ 1 = Strongly disagree

- ☐ 2 = Disagree
- ☐ 3 = Undecided
- ☐ 4 = Agree
- ☐ 5 = Strongly agree
- ☐ 0 = Don't know

If you want to add more details to your answer, please mention them:
…………………………………………………………………………………………………
…………………………………………

11/In your opinion, does the health care professional have some responsibility in these inequalities of access to the kidney transplant?

- ☐ 1 = Strongly disagree
- ☐ 2 = Disagree
- ☐ 3 = Undecided
- ☐ 4 = Agree
- ☐ 5 = Strongly agree
- ☐ 0 = Don't know

If you want to add more details to your answer, please mention them:
…………………………………………………………………………………………………
…………………………………………

12/Finally, do you think there may be other criteria that may hinder access to registration on the kidney transplant waiting list?

- ☐ 1 = Strongly disagree
- ☐ 2 = Disagree
- ☐ 3 = Undecided
- ☐ 4 = Agree
- ☐ 5 = Strongly agree
- ☐ 0 = Don't know

If you want to add more details to your answer, please mention them:
…………………………………………………………………………………………………
…………………………………………
