# Supplementary material for: Perception of social inequities in the access to the kidney transplant waiting list by nephrology trainees: a national survey
Source: BMC Nephrol. 2022 Dec 8;23:394. doi: 10.1186/s12882-022-03017-w (PMC9733200; doi:10.1186/s12882-022-03017-w)
Supplement: Supplementary file 2 — Additional file 2. Graphical representation of the item responses. [file 12882_2022_3017_MOESM2_ESM.pdf]

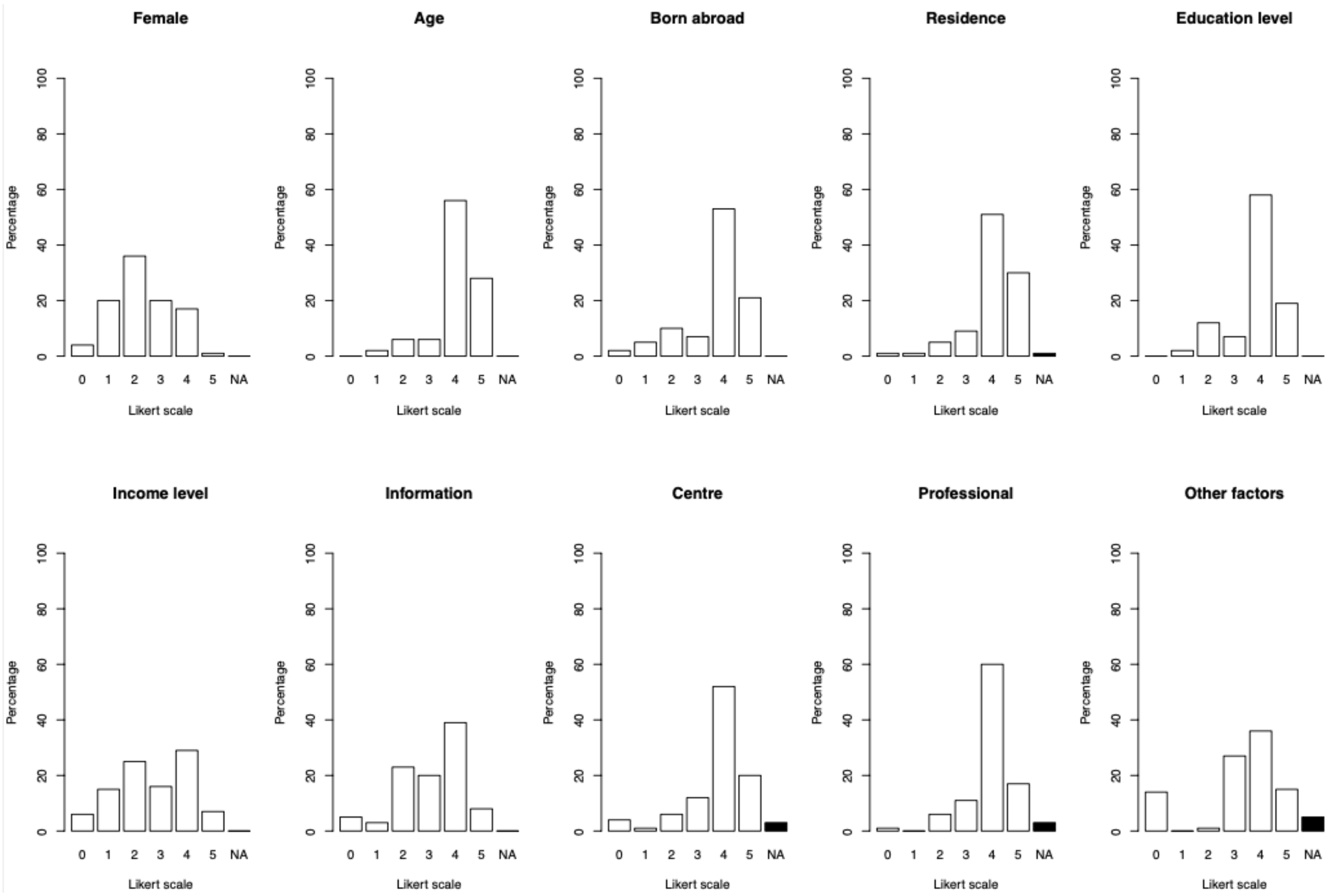

Additional file 2. Graphical representation of the item responses

10 items answered using the Likert scale of 6 points: don't know (0), strongly disagree (1), disagree (2), undecided (3), agree (4), strongly agree (5). Information: centre provision to adapt the information, Professional: health care professional
