# Supplementary material for: Perception of social inequities in the access to the kidney transplant waiting list by nephrology trainees: a national survey
Source: BMC Nephrol. 2022 Dec 8;23:394. doi: 10.1186/s12882-022-03017-w (PMC9733200; doi:10.1186/s12882-022-03017-w)
Supplement: Supplementary file 3 — Additional file 3. Principal component analysis. [file 12882_2022_3017_MOESM3_ESM.pdf]

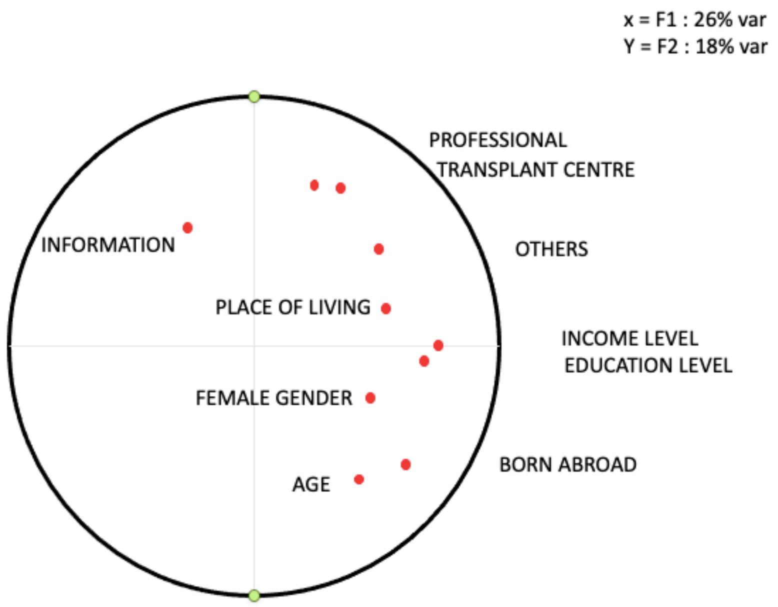

Additional file 3. Principal component analysis

Forty-four percent (26% + 18%) of the initial variance of the data is represented in the picture. INFORMATION: centre provision to adapt the information, PROFESSIONAL: health care professional, OTHERS: other factors
