# Supplementary material for: Perception of social inequities in the access to the kidney transplant waiting list by nephrology trainees: a national survey
Source: BMC Nephrol. 2022 Dec 8;23:394. doi: 10.1186/s12882-022-03017-w (PMC9733200; doi:10.1186/s12882-022-03017-w)
Supplement: Supplementary file 4 — Additional file 4. Hierarchical clustering. [file 12882_2022_3017_MOESM4_ESM.pdf]

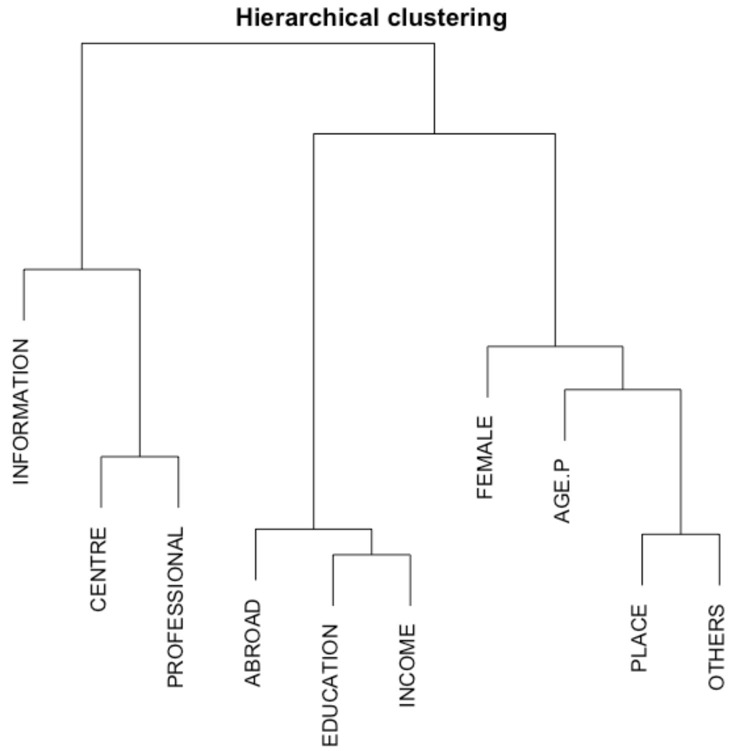

Additional file 4. Hierarchical clustering
INFORMATION: centre provision to adapt the information, CENTRE: transplant centre, PROFESSIONAL: health care professional, ABROAD: born abroad, EDUCATION: education level, INCOME: income level, FEMALE: female sex, AGE. P: age of the patient, PLACE: living place, OTHERS: other factors
