# Supplementary material for: Perception of social inequities in the access to the kidney transplant waiting list by nephrology trainees: a national survey
Source: BMC Nephrol. 2022 Dec 8;23:394. doi: 10.1186/s12882-022-03017-w (PMC9733200; doi:10.1186/s12882-022-03017-w)
Supplement: Supplementary file 5 — Additional file 5. Scree plots. [file 12882_2022_3017_MOESM5_ESM.pdf]

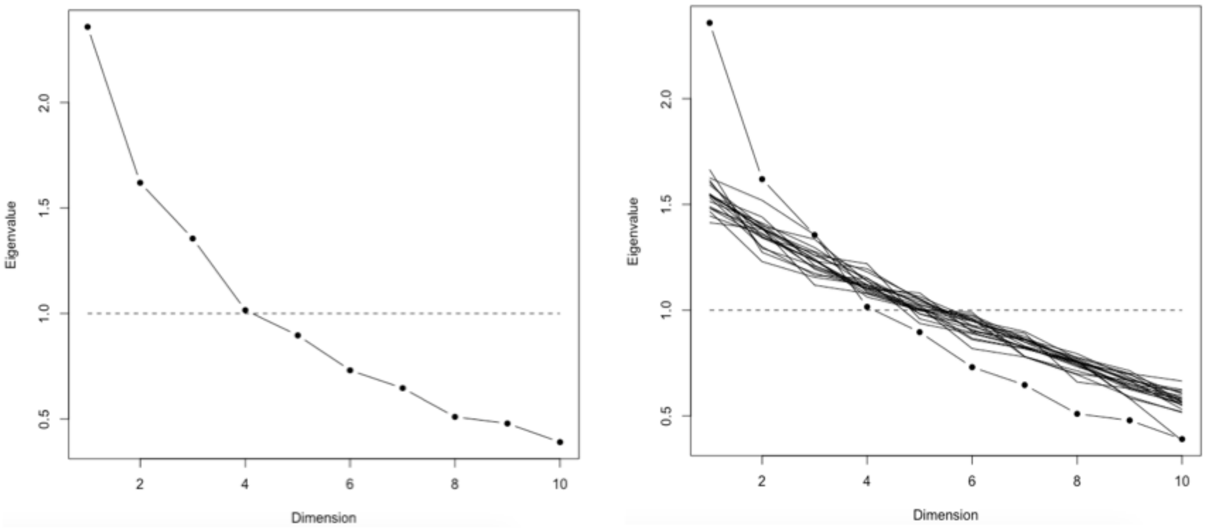

Additional file 5. Scree plots
Left side: scree plot for 10 items (all of the questions, except the variables male sex and the multiple choice questions on specific categories of person)
Right side: Scree plot for the same 10 items, with a parallel analysis
